# Complex regulation of neutrophil-derived MMP-9 secretion in central nervous system tuberculosis

**DOI:** 10.1186/s12974-017-0801-1

**Published:** 2017-02-07

**Authors:** Catherine W. M. Ong, Przemyslaw J. Pabisiak, Sara Brilha, Poonam Singh, Federico Roncaroli, Paul T. Elkington, Jon S. Friedland

**Affiliations:** 1 Section of Infectious Diseases and Immunity, Hammersmith Campus, Imperial College London, 8th floor Commonwealth Building, Du Cane Road, London, W12 0NN UK; 2 Division of Infectious Diseases, Department of Medicine, Yong Loo Lin School of Medicine, National University of Singapore, Singapore, Singapore; 3 Department of Histopathology, Hammersmith Campus, Imperial College London, London, UK; 4 Division of Neuroscience and Experimental Psychology, University of Manchester, Manchester, UK; 5 NIHR Respiratory Biomedical Research Unit, Faculty of Medicine, University of Southampton, Southampton, UK

**Keywords:** Tuberculosis, Immunopathology, Neutrophils, Matrix metalloproteinase

## Abstract

**Background:**

Central nervous system tuberculosis (CNS-TB) may be fatal even with treatment. Neutrophils are the key mediators of TB immunopathology, and raised CSF matrix metalloproteinase-9 (MMP-9) which correlates to neutrophil count in CNS-TB is associated with neurological deficit and death. The mechanisms by which neutrophils drive TB-associated CNS matrix destruction are not clearly defined.

**Methods:**

Human brain biopsies with histologically proven CNS-TB were stained for neutrophils, neutrophil elastase, and MMP-9. Neutrophil MMP-9 secretion and gene expression were analyzed using Luminex and real-time PCR. Type IV collagen degradation was evaluated using confocal microscopy and quantitative fluorescent assays. Intracellular signaling pathways were investigated by immunoblotting and chemical inhibitors.

**Results:**

MMP-9-expressing neutrophils were present in tuberculous granulomas in CNS-TB and neutrophil-derived MMP-9 secretion was upregulated by *Mycobacterium tuberculosis* (*M.tb*). Concurrent direct stimulation by *M.tb* and activation via monocyte-dependent networks had an additive effect on neutrophil MMP-9 secretion. Destruction of type IV collagen, a key component of the blood-brain barrier, was inhibited by neutralizing neutrophil MMP-9. Monocyte-neutrophil networks driving MMP-9 secretion in TB were regulated by MAP-kinase and Akt-PI_3_ kinase pathways and the transcription factor NF-kB. TNFα neutralization suppressed MMP-9 secretion to baseline while dexamethasone did not.

**Conclusions:**

Multiple signaling paths regulate neutrophil-derived MMP-9 secretion, which is increased in CNS-TB. These paths may be better targets for host-directed therapies than steroids currently used in CNS-TB.

## Background

Despite treatment, central nervous system tuberculosis (CNS-TB) often results in severe neurological deficit and frequent death. Dexamethasone, a recommended adjunct to CNS-TB treatment, improves mortality but does not affect long-term neurological outcome [1, 2]. This has been attributed to innate host factors such as the LTA_4_ genotype [3], underscoring the role of host-driven immunopathology in this devastating infection. Newer interventions for CNS-TB are urgently needed to improve both morbidity and mortality, particularly in the era of rising drug resistance. Host-directed therapies may be an attractive therapeutic strategy [4].

Neutrophils are emerging mediators of TB pathology. These early sentinels to *Mycobacterium tuberculosis* infection play the key roles in TB inflammation [5, 6]. Raised neutrophils are present in CNS-TB in the setting of both HIV-negative and HIV-associated immune reconstitution inflammatory syndrome and increased neutrophil-associated mediators such as S100A calcium-binding protein correspond to the degree of inflammation [7–9]. However, the mechanisms by which neutrophils cause neuroinflammation in CNS-TB are not defined.

The development of severe neurological deficits may be caused by local CNS tissue destruction. Tissue damage may be driven by the host immune cells recruited to the CNS such as neutrophils and macrophages [10, 11], following disruption to the blood-brain barrier (BBB). These cells secrete matrix metalloproteinases (MMPs), zinc-containing proteases which degrade extracellular matrix fibrils crucial for the integrity of the BBB [12]. MMPs are inhibited by specific tissue inhibitors of metalloproteinases (TIMPs). We and others found increased MMP expression in CNS-TB and raised MMP concentrations were associated with neurological deficit and death [7, 8, 13]. In addition, mediators including TNFα which is the key in the defense against mycobacteria and whose blockade leads to reactivation of TB [14, 15], drive MMP secretion from the host cells including the neutrophils and epithelial cells and may have a role in CNS-TB immunopathology [16, 17]. Investigating mechanisms by which MMPs result in tissue damage may be the key in understanding the pathogenesis of CNS neuroinflammation caused by agents such as *M.tb*.

We hypothesized that neutrophils drive matrix destruction in CNS-TB. As humans are the primary host of *M.tb*, we examined the brain biopsies of patients with proven CNS-TB and investigated our findings in a human cellular model. Neutrophils expressing MMP-9 are present in CNS tuberculous granulomas, and *M.tb* infection increased neutrophil MMP-9 secretion and gene expression. Neutrophil-derived MMP-9 was functionally active and caused type IV collagen destruction, which was reversed by neutralizing MMP-9. We demonstrate that mitogen activated protein-kinase (MAP-kinase) and the phosphoinositide-3 (PI_3_) kinase-Akt pathways regulated neutrophil MMP-9 secretion in monocyte-dependent intercellular networks. Neutralizing TNFα suppressed neutrophil MMP-9 to baseline, while dexamethasone did not, which may partly explain the limited benefit of steroids in patients. Taken together, our findings suggest that host-directed therapy targeting MMP-9 secretion may have a potential to limit immunopathology in CNS-TB.

## Methods

### Reagents and antibodies

Dexamethasone was from Sigma. Helenalin and SC-514 were from Merck Biochemicals. SB203580, PD98059, and LY294002 were from Enzo Life Sciences. Goat anti-human TNFα was from PeproTech. Mouse anti-human MMP-9, rabbit anti-*M.tb*, and goat anti-rabbit IgG Cy5 were from Abcam. Rabbit anti-human phospho-Akt, total-Akt, phospho-p38, total-p38, phospho-ERK, total-ERK, phospho-JNK, total-JNK, and goat anti-rabbit HRP linked were from Cell Signaling Technology. Rabbit anti-human neutrophil elastase was from Dako, and mouse anti-human MMP-9 was from Millipore.

### Recruitment of patients and controls

#### Immunohistochemistry

The paraffin blocks of five surgical samples from immunocompetent patients with CNS *M.tb* infection were retrieved from the files of the Department of Histopathology at Imperial College Healthcare Trust, London. All specimens contained leptomeninges, cortex, and subcortical white matter and showed typical necrotizing granulomas and acid-fast bacilli identified with the Ziehl-Neelsen stain. Sections of caecal appendix with acute inflammation were used as positive control for MMP-9 immunoreactivity. To prove the specificity of primary antibodies directed against MMP-9 and elastases, we used sections from the frontal lobe of five post-mortem brain with only mild aging-related changes. The appendix and brains were also retrieved from the files of the Department of Histopathology at Imperial College. Samples were annonymized for the purpose of this study. The cases were investigated using immunoperoxidase immunohistochemistry with antibodies directed against MMP-9 (Abcam; 1: 200) and neutrophil elastase (Dako, clone NP57; 1/100). Immunostains with omission of the primary antibody were performed as negative controls. Five-micron sections were cut from each block, dewaxed in xylene, and rehydrated in decreasing alcohols to distilled water. Endogenous peroxidase activity was blocked in 0.3% hydrogen peroxide in methanol for 30 min. For antigen retrieval, sections were steamed for 20 min in 0.01 M citrate, pH 6.5, and then gently cooled in water. In order to block nonspecific binding of the primary antibody, sections were incubated with 10% normal goat serum for 10 min (Vector Laboratories, Burlingame, California). The primary antibodies were applied overnight at 4 °C. After incubation, they were washed for three times in PBS for 10 min each. Staining was visualized using the VECTASTAIN Elite ABC Kit (Vector Laboratories) following the manufacturer instructions using 2 ng/ml 3,3′-diaminobenzidine and 0.0075% hydrogen peroxide in PBS as chromogen.

### *M.tb* culture

*M.tb* H37Rv was cultured in supplemented Middlebrook 7H9 medium (BD Biosciences). For infection experiments, mycobacteria were used at mid-logarithmic growth at an optical density of 0.60 (Biowave cell density meter; WPA).

### Cell culture and stimulation

The whole blood from healthy volunteers were drawn in preservative-free heparin and mixed with equal volumes of 3% dextran saline to remove erythrocytes. Neutrophils were isolated from the resulting cell suspension using Ficoll-Paque density centrifugation and three rounds of hypotonic lysis. Neutrophil purity was over 95% by FACS and viability >99% by trypan blue assay. In some experiments, neutrophils were pre-incubated with specific inhibitors/agents as indicated for 30 min unless otherwise stated. In all experiments involving live *M.tb* H37Rv, tissue culture medium was sterile filtered through 0.2 μm Anopore membranes (Millipore) before removing from the containment level 3 tuberculosis laboratory. All experiments were performed using 4 h incubations unless otherwise stated.

Primary human blood monocytes were prepared from donor leukocyte cones from healthy donors (National Blood Transfusion Service, UK). After density gradient centrifugation (Ficoll-Paque) followed by adhesion purification, monocyte purity was over 95% by FACS analysis. Monocytes were infected with *M.tb* at a multiplicity of infection (MOI) of 1. After incubation at 37 °C for 24 h, conditioned medium was harvested and was termed CoMTB. Media from uninfected monocytes were termed CoMCont.

### ELISAs for TIMP-1/2, MPO, and NGAL

TIMP-1 and -2 concentrations were measured using the Duoset ELISA Development System (R&D Systems) and detected a minimum of 31.2 pg/ml for both. Myeloperoxidase (MPO) was measured using the human MPO Quantikine ELISA Kit (R&D Systems) which had a minimum detection limit of 0.1 ng/ml. Neutrophil gelatinase-associated lipocalin (NGAL) was measured using the human NGAL ELISA Kit (BioPorto Diagnostics) which had minimum detection limit of 1.6 pg/ml.

### Luminex array

MMP-8 and -9 concentrations were analyzed by Fluorokine MultiAnalyte Profiling Kit according to manufacturer’s protocol (R&D Systems) on the Luminex 200 platform (Bio-Rad). The minimum level of detection for MMP-8 and -9 was 110 and 65 pg/ml, respectively.

### DQ collagen degradation assay

Type IV collagen degradation was assessed using the EnzChek ® Gelatinase/Collagenase Assay kit (Molecular Probes). Samples were activated with 2 mM of 4-amino-phenyl mercuric acetate (APMA) for 1 h at 37 °C. Eighty microliters of reaction buffer or inhibitor (mouse anti-human MMP-9) were added with 20 μL of DQ collagen (Invitrogen) at a final concentration of 25 μg/ml. The activated samples were subsequently added, and activity was detected at specified times using a fluorometer (FLUOstar Galaxy).

### Immunoblotting

Pelleted neutrophils infected with *M.tb* or stimulated with CoMTB were mixed with SDS lysis buffer. The samples were run on the NuPAGE® 4-12% Bis-Tris gels with SDS Running buffer (Invitrogen). Protein was transferred onto a nitrocellulose membrane (GE Healthcare). Primary antibody was diluted in 5% BSA/0.1% Tween and incubated overnight at 4 °C with agitation. Secondary antibody was added diluted in blocking buffer. Luminescence was demonstrated with ECL Substrate Reagent (Amersham Science) according to manufacturer’s instructions and exposing the membrane to Hyperfilm ECL. Densitometric analysis was performed using ImageJ 1.43U (NIH, USA).

### Real-time PCR

Total RNA was extracted from 2 × 10^6^ neutrophils using the RNeasy Mini Kit (Qiagen). Quantitative real-time RT-PCR was performed using the One-Step RT-PCR Master Mix (Qiagen) according to manufacturer’s instruction on a Stratagene Mx3000P platform using 5–10 μg per sample. MMP-9 (forward primer 5′-AGGCGCTCATGTACCCTATGTAC-3′, reverse primer 5′-GCCGTGGCTCAGGTTCA-3′, Probe 5′-FAM-CATCCGGCACCTCTATGGTCCTCG-TAMRA-3′) with glyceraldehyde 3-phosphate dehydrogenase (GAPDH) (Forward primer 5′-CGCTTCGCTCTCTGCTCCT-3′, reverse primer 5′-CGACCAAATCCGTTGACTCC-3′, probe 5′-HEX-CGTCGCCAGCCGAGCCACAT-TAMRA-3′) was analyzed in parallel. To accurately determine the quantitative change in RNA, standard curves were prepared from plasmids subjected to real-time PCR as above. MMP-9 data were normalized to GAPDH detected in the same sample.

### Immunofluorescence microscopy

Permanox chamber slides (Nunc Lab-Tek) were coated with 25 μg/ml of DQ collagen for 30 min. The samples were then fixed with 4% paraformaldehyde for 30 min and permeabilized with 0.5% saponin for 10 min. The cells were washed before blocking with 10% human AB serum with 2.5% BSA and 0.05% saponin. Primary antibodies were added overnight. Chamber slides were washed prior to the addition of secondary antibodies. The chambers were subsequently removed from the slide, and saline was added. Images were captured using Leica confocal microscope (Leica TCS SP5) and processed using Leica LAS AF Lite 2.6.0 (Leica Microsystems, Germany) and ImageJ 1.43U (NIH, USA).

#### Flow cytometry

Cell viability was assessed by staining neutrophils with annexin V and propidium iodide using Annexin V-FITC Apoptosis Detection Kit (eBioscience, Affymetrix, California, USA) and LIVE/DEAD Fixable Stain Kit (Invitrogen). Neutrophils were stimulated with 200 ng/ml staurosporine to induce apoptosis, and this was used as a positive control for all experiments. Annexin V was detected on the FL-1 channel with propidium iodide and LIVE/DEAD Fixable Dead Cell Stain Kit on FL-3. A total of 50,000 events were gated and analyzed on BD FACSCalibur flow cytometer using CellQuest. Data was analyzed using FlowJo 7.6.5 (Tree Star).

### Statistical analyses

Data were analyzed using GraphPad Prism (version 5.04, GraphPad Software). Data are expressed as mean ± s.d. unless stated otherwise. All experiments are performed in biological triplicates on at least two separate occasions. Multiple intervention experiments are compared with one-way ANOVA followed by Tukey’s post-test correction, while continuous variables between two sets of data are assessed using two-tailed Mann-Whitney *U* test. Spearman’s rank correlation tests are used for correlation analyses. *P* values of less than 0.05 are taken as statistically significant.

## Results

### Neutrophils express MMP-9 in response to direct *M.tb* infection or stimulation by *M.tb*-driven monocyte-dependent networks and in patients with CNS-TB

**Fig. 1:**
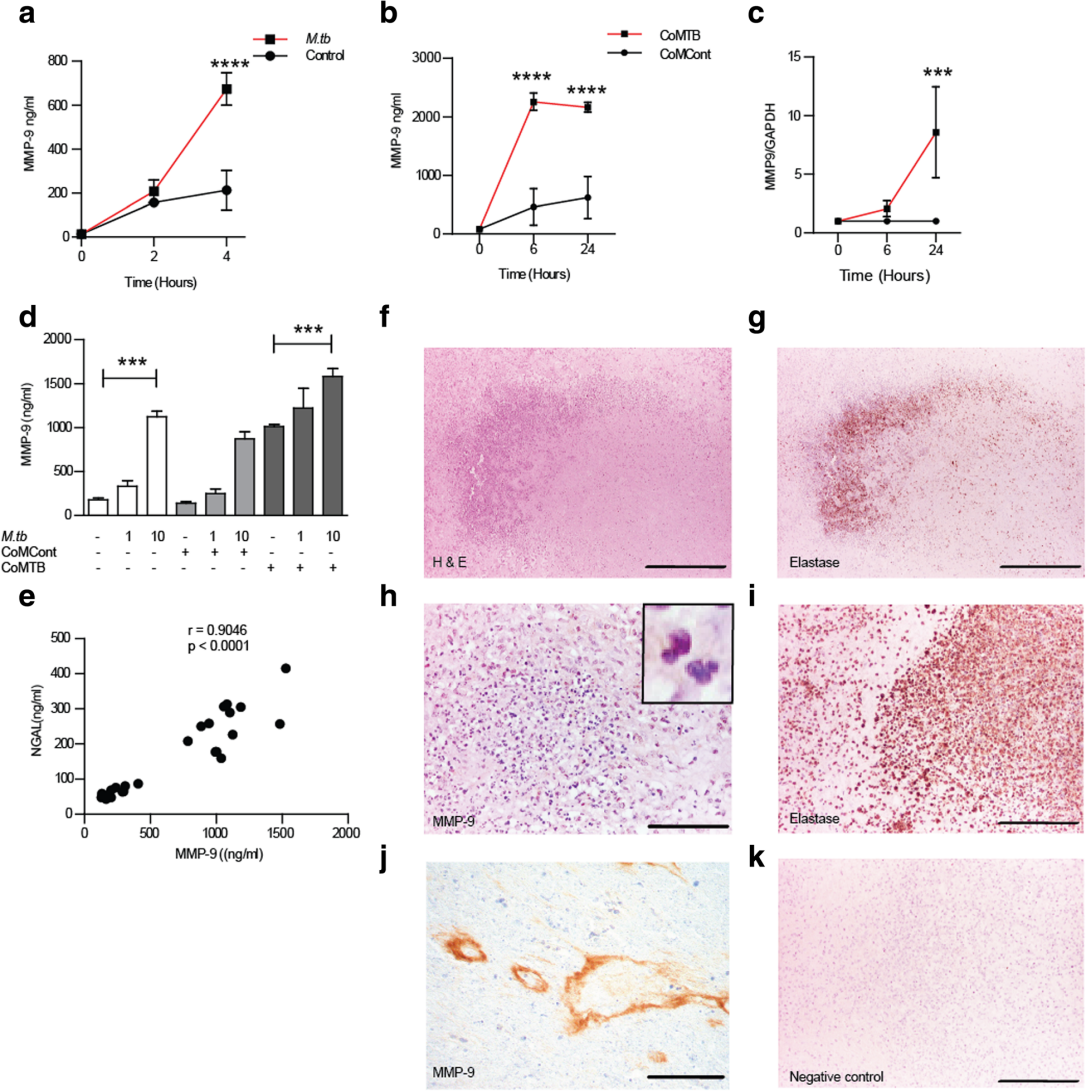
Neutrophils secrete MMP-9 and are present in CNS-TB. **a** *M.tb* infection with MOI of 1 upregulates neutrophil MMP-9 secretion. **b** Neutrophils from healthy volunteers secrete MMP-9 in response to CoMTB. **c** Kinetics of Neutrophil MMP-9 gene expression after CoMTB stimulation. **d** *M.tb* and CoMTB stimulation have an additive effect on neutrophil MMP-9 secretion. **e** NGAL and MMP-9 concentrations closely correlate despite being located in different neutrophil granules. **f**–**h** Neutrophils expressing MMP-9 are present in CNS TB granulomas and present in vessel walls and perivascular interstitial space. **f** H&E, *scale bar* 250 μm. **g** Elastase immunoperoxidase, *scale bar* 250 μm and **h** MMP-9 immunoperoxidase, ×20 magnification, *scale bar* 50 μm. *Inset* shows neutrophils within lesions (×40). Positive control on acute appendicitis with **i** elastase, *scale bar* 50 μm and **j** MMP-9 on blood vessel wall and interstitium, *scale bar* 200 μm. **k** Negative control with omission of primary antibodies, *scale bar* represents 100 μm. *Bars* represent mean ± s.d and represent at least two independent experiments performed in triplicate. *P < 0.05; **P < 0.01; ***P < 0.001; ****P < 0.0001

First, we investigated MMP-9 secretion from primary human neutrophils in TB. Neutrophil MMP-9 secretion increased over 4 h in response to *M.tb* infection (Fig. 1a, *P* < 0.0001). By 24 h, more than 80% of neutrophils were non-viable after infection by flow cytometry staining of annexin V and propidium iodide. Intercellular networks are crucial in TB [18], so we evaluated the effect of cross talk between neutrophils and monocytes [19] by using conditioned media from monocytes infected by *M.tb* (CoMTB). CoMTB stimulation caused significant upregulation of MMP-9 secretion by 3.5-fold compared to CoMCont (Fig. 1b, *P* < 0.0001). CoMTB increased neutrophil MMP-9 gene expression 8.6-fold (Fig. 1c, *P* < 0.001). As direct *M.tb* infection and intercellular network interactions occur simultaneously in TB, we investigated the effect of concurrent *M.tb* and CoMTB stimulation of neutrophils and demonstrated an additive effect on MMP-9 secretion (Fig. 1d). We also analyzed the secretion of NGAL which can restrict the growth of *M.tb* [20]. Neutrophil MMP-9 secretion closely correlated with the secretion of NGAL in response to both *M.tb* and CoMTB stimulation (Fig. 1e; *r* = 0.9, *P* < 0.0001).

To determine whether neutrophils express MMP-9 at the site of disease in patients with CNS-TB, we analyzed specimens from patients who underwent diagnostic biopsies. Polymorphonuclear neutrophils were observed around the granuloma on H & E staining (Fig. 1f). Neutrophil accumulation was confirmed by specific positive staining for neutrophil elastase and MMP-9 (Fig. 1g, h). MMP-9 was also seen in the blood vessel wall and perivascular interstitium (Fig. 1j). The *M.tb*-infected brain tissue with secondary antibody alone showed no immunoreactivity (Fig. 1k).

### *M.tb*-infected neutrophils degrade basement membrane matrix

**Fig. 2:**
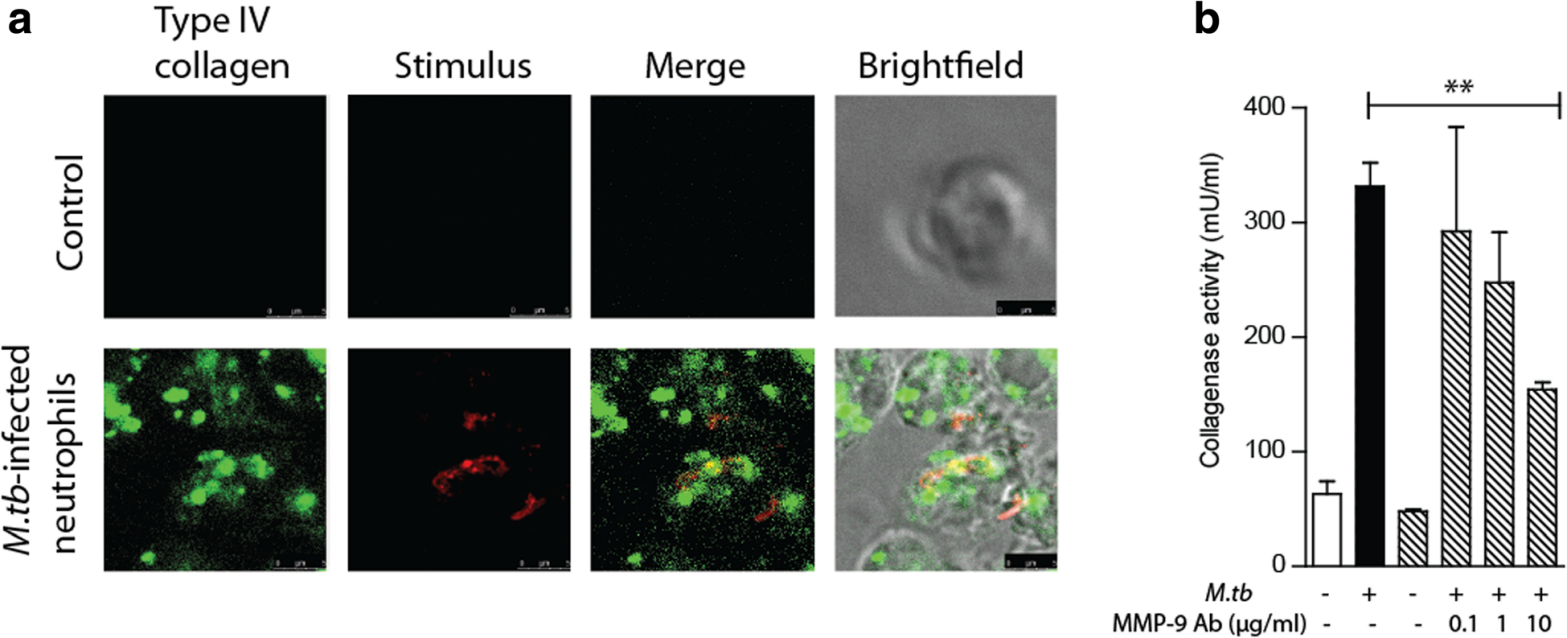
Collagen is degraded by neutrophils stimulated with *M.tb* in an MMP-9 dependent manner. **a** Confocal microscopy of Type IV DQ collagen coated slides demonstrating collagen degradation in the presence of *M.tb* stimulation. DQ collagen fluorescence increases after degradation. **b** Type IV collagenase activity is inhibited by MMP-9 neutralizing antibody. *Bars* represent mean ± s.d and represent two independent experiment performed in triplicate. **P < 0.01

Type IV collagen is the main extracellular matrix fibril in the basement membrane of the BBB [21]. Therefore, we assessed the functional activity of neutrophil MMP-9 on this key structural protein. Confocal microscopy demonstrated type IV collagen degradation at the neutrophil-collagen interface in *M.tb*-infected neutrophils, but not uninfected neutrophils (Fig. 2a). Increased collagenase activity after *M.tb* infection was confirmed by a quantitative fluorescence assay (Fig. 2b). Pre-treatment of *M.tb*-stimulated neutrophil supernatants with neutralizing anti-MMP-9 antibody caused a dose-dependent inhibition of collagenase activity with a maximal effect at 10 μg/ml (Fig. 2b, *P <* 0.01), demonstrating that this collagenolysis was MMP-9 dependent.

### Neutrophil MAP-kinase pathways regulate CoMTB-induced MMP-9 secretion

**Fig. 3:**
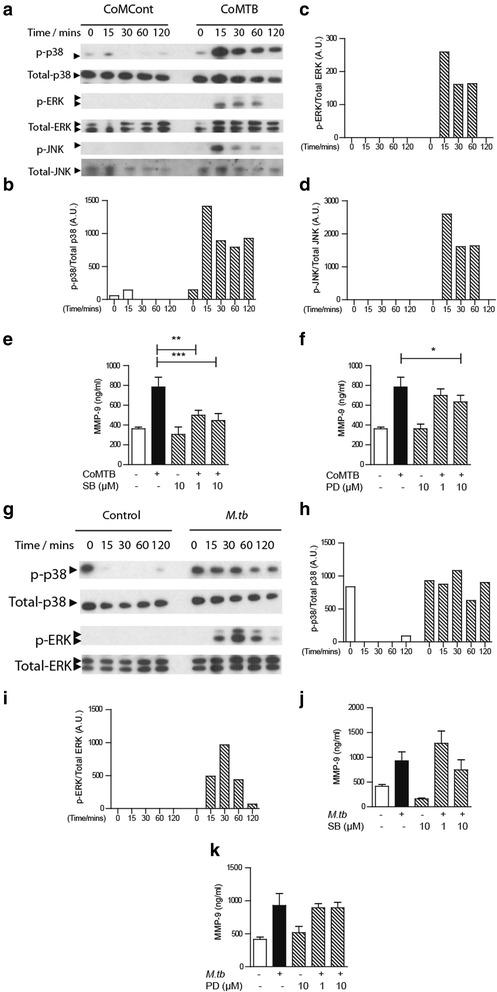
MAP-kinase pathways are activated in neutrophils in TB and regulate MMP-9 secretion. **a** Representative immunoblots of p-38, ERK, and JNK MAP kinases in neutrophils stimulated with CoMTB. CoMTB increases phosphorylation of all pathways from 15 min. Densitometric analysis of response to CoMCont and CoMTB stimulation of **b** phospho-p38 to total p38, **c** phospho-ERK to total ERK, and **d** phospho-JNK to total JNK. **e** CoMTB-stimulated MMP-9 secretion is suppressed by p-38 inhibitor SB203580. **f** MMP-9 secretion with CoMTB is inhibited by ERK inhibitor PD98059. **g** Representative immunoblots to detect p-38, ERK MAP kinases in neutrophils stimulated with *M.tb*. Densitometric analysis of response to control (PBS) and *M.tb* infection of **h** phospho-p-38 to total p-38 and **i** phospho-JNK to total JNK. **j** Pre-incubation with p-38 inhibitor SB does not suppress MMP-9 secretion in *M.tb*-infected neutrophils. **k** Pre-incubation with ERK inhibitor PD does not affect MMP-9 secretion by *M.tb*-stimulated neutrophils. All chemical inhibitor experiments were performed by pre-incubating neutrophils for 30 min before stimulating for 4 h. *Bars* represent mean ± s.d and are representative of at least two independent experiments performed in triplicate. *P < 0.05; **P < 0.01; ***P < 0.001

Next, we investigated the neutrophil intracellular pathways regulating MMP-9 secretion. As the MAP-kinase pathway regulates MMPs in other CNS cell types [22], we evaluated components of the pathway following CoMTB stimulation of neutrophils. Immunoblotting revealed phosphorylation of p38, ERK, and JNK with corresponding increase of phospho-kinase to total kinase ratio after CoMTB stimulation (Fig. 3a–d). Using targeted inhibitors against p38 and ERK [23], we demonstrated a 48% MMP-9 inhibition with 10 μM of p38 inhibitor SB203580 (Fig. 3e, *P* < 0.001) and a 15% inhibition with 10 μM of ERK inhibitor PD98059 compared to neutrophil stimulation with CoMTB alone (Fig. 3f, *P* < 0.05). With live virulent *M.tb* stimulation, we demonstrated phosphorylation of the p38 and ERK components of the MAP kinase pathway (Fig. 3g–i). However, in contrast to CoMTB stimulation, pre-incubation of neutrophils with p38 inhibitor or ERK inhibitors did not suppress MMP-9 secretion after *M.tb* infection (Fig. 3j, k).

### The Akt pathway is activated and regulates MMP-9 secretion after CoMTB stimulation but not direct *M.tb* infection

**Fig. 4:**
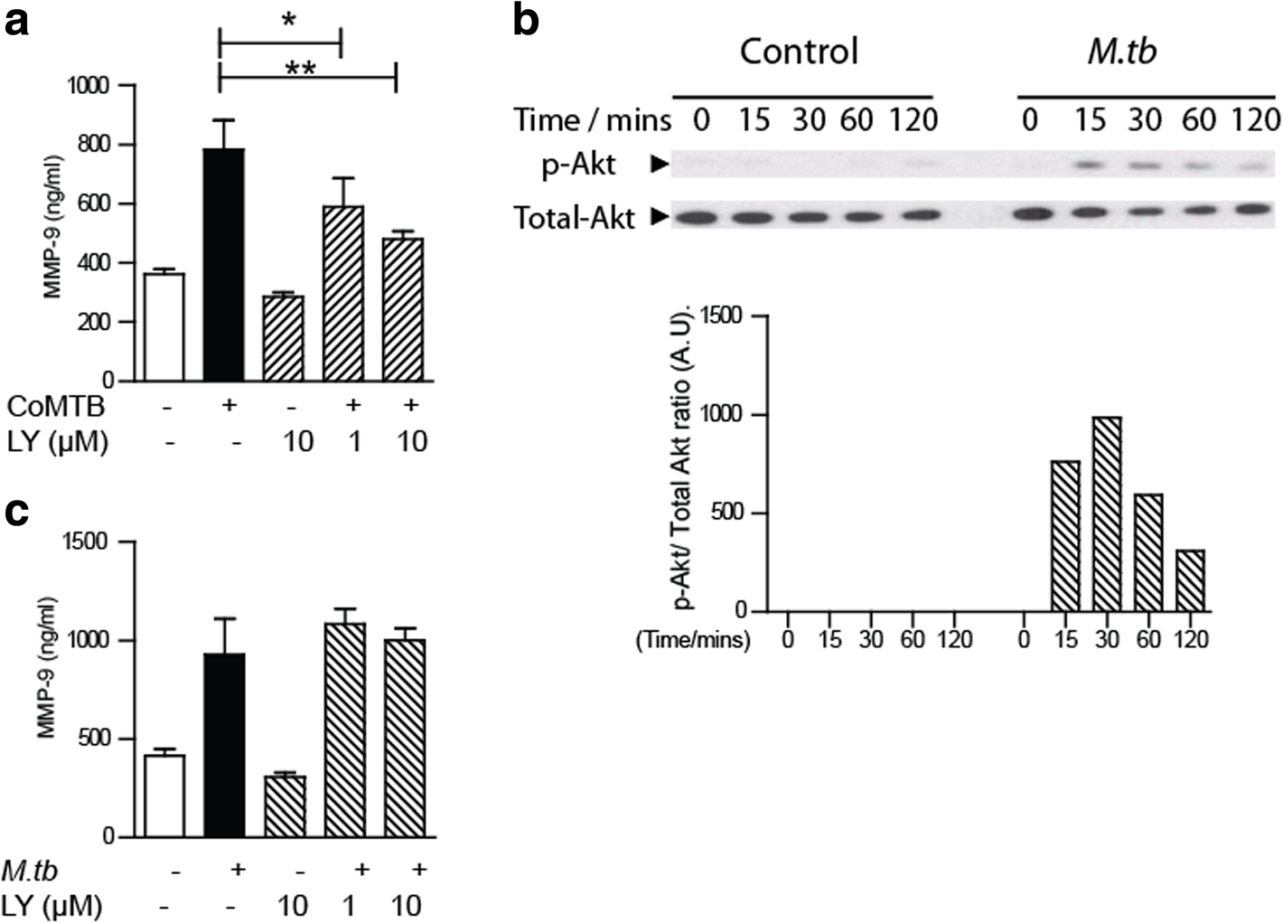
The Akt pathway regulates neutrophil MMP-9 secretion in TB. **a** Pre-incubation with PI3-kinase inhibitor LY294002 suppresses MMP-9 secretion with CoMTB. **b** Representative immunoblots to detect p-Akt in neutrophils infected with *M.tb* show increased phosphorylation with corresponding densitometric analysis. **c** Pre-incubation with PI3-kinase inhibitor LY does not affect MMP-9 secretion after *M.tb* infection. *Bars* represent mean ± s.d and are representative of at least two independent experiments performed in triplicate. *P < 0.05; **P < 0.01

The PI_3_ kinase-Akt pathway regulates bronchial epithelial cell-derived MMPs in TB [24]. We had previously shown that Akt was phosphorylated in the presence of CoMTB [6] and therefore investigated if this path also regulates neutrophil MMP-9 secretion. Pre-treating neutrophils with PI_3_ kinase inhibitor LY294002 showed a dose-dependent suppression of MMP-9 of 1.6-fold with CoMTB stimulation (Fig. 4a, *P* < 0.01). Similarly with *M.tb*, Akt was phosphorylated, indicating activation (Fig. 4b). However, PI_3_kinase inhibitor LY294002 did not inhibit neutrophil MMP-9 secretion with *M.tb* stimulation (Fig. 4c).

### Transcription factor NF-kB regulates neutrophil MMP-9 in TB

**Fig. 5:**
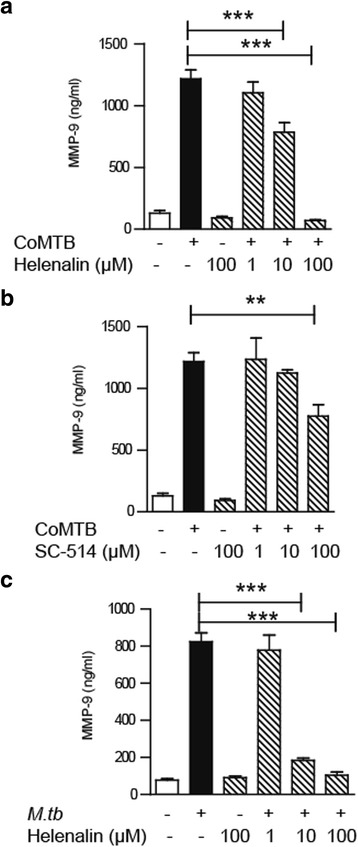
Neutrophil MMP-9 secretion is regulated by NF-kB. **a** Pre-incubation with helenalin inhibits MMP-9 secretion after CoMTB stimulation. **b** Pre-incubation with IKK2 inhibitor SC-514 inhibits MMP-9 secretion with CoMTB stimulation. **c** Pre-incubation with NF-kB inhibitor helenalin inhibits MMP-9 secretion after *M.tb* infection. *Bars* represent mean ± s.d and are representative of at least two independent experiments performed in triplicate. **P < 0.01; ***P < 0.001

NF-kB is one of the crucial transcription factors driving inflammation in TB [6]. We evaluated if NF-kB regulated neutrophil MMP-9 secretion by using NF-kB p65 subunit inhibitor helenalin (IC_50_ 10–50 μM) and IKK_2_ inhibitor SC-514 (IC_50_ 3–12 μM). In the presence of CoMTB stimulation, we demonstrated a dose-dependent suppression of neutrophil MMP-9 to baseline with helenalin (Fig. 5a, *P* < 0.001). Suppression of neutrophil MMP-9 secretion was similarly achieved with SC-514 (Fig. 5b, *P* < 0.01). Pre-treating neutrophils with helenalin before *M.tb* infection also resulted in a decrease in neutrophil MMP-9 secretion, which was maximal at a dose of 100 μM (Fig. 5c, *P* < 0.001), but at higher concentrations, neutrophil viability declined markedly to less than 70% by FACS. Therefore, NF-kB regulates neutrophil MMP-9 secretion for both direct infection and intercellular networks.

### Anti-TNFα suppresses neutrophil MMP-9 secretion, but dexamethasone does not

**Fig. 6:**
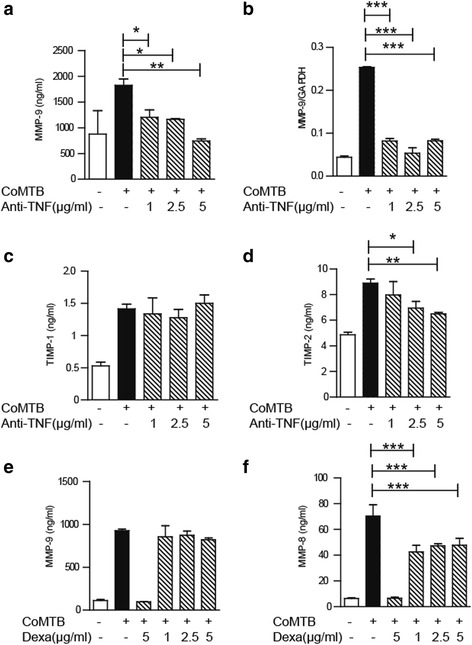
Anti-TNFα suppresses neutrophil MMP-9 secretion, but dexamethasone does not. **a** MMP-9 secretion is inhibited by anti-TNFα pre-treatment of neutrophils. CoMTB was incubated with anti-TNFα antibodies for 1 h before neutrophil stimulation. **b** Anti-TNFα inhibits MMP-9 gene expression. **c** TIMP-1 secretion is not affected by anti-TNFα. **d** TIMP-2 secretion is reduced by anti-TNFα. **e** MMP-9 secretion is not affected by dexamethasone. **f** Dexamethasone inhibits neutrophil MMP-8 secretion. *Bars* represent mean ± s.d and are representative of at least two independent experiments performed in triplicate. *P < 0.05; **P < 0.01; ***P < 0.001

Neutrophil MMPs are raised in CNS-TB [8], and anti-TNFα antagonists have been used to treat severe paradoxical reaction in CNS-TB with improvement to neurological outcome [25, 26]. We investigated the effect of TNFα neutralizing antibodies on neutrophil MMP secretion. Pre-incubating CoMTB with anti-TNFα prior to stimulating human neutrophils resulted in suppression of MMP-9 secretion and gene expression which was maximal at a dose of 5 μg/ml (Fig. 6a, b; *P* < 0.01 and *P* < 0.001, respectively). TIMP-1 secretion was unaffected by anti-TNFα (Fig. 6c) while TIMP-2 secretion was slightly suppressed in a dose-dependent manner (Fig. 6d, *P* < 0.01). Dexamethasone is an established adjunct treatment for patients with CNS-TB that improves mortality but does not significantly affecting neurological outcome [1], so we investigated the effect of dexamethasone on neutrophil MMP secretion. MMP-9 secretion was unaffected by dexamethasone at a dose of 5 μg/ml, similar to the dose used in clinical trials (Fig. 6e). However, CoMTB-driven neutrophil MMP-8 secretion was inhibited by 32% (Fig. 6f, *P* < 0.001). We also evaluated if TNFα drove neutrophil MMP-9 secretion in *M.tb* infection, but this was not altered.

## Discussion

CNS-TB is a devastating infection driven by an excessive host immune response to infection and results in neurological morbidity and death. Our findings reveal a mechanism by which neutrophils contribute to tissue destruction in the brain, further supporting the role of the granulocyte in CNS-TB pathogenesis [8, 9, 27]. We have demonstrated that neutrophils within granulomas express MMP-9 in the brain biopsies from patients with proven CNS-TB, further highlighting the role of MMPs in pathology of TB granulomas [28]. Neutrophils secrete MMP-9 in response to both direct *M.tb* infection and monocyte-dependent networks in CNS-TB. The combination of the two stimuli has an additive effect to MMP-9 secretion, which is partially driven by TNFα, as demonstrated by a decrease in MMP-9 with the addition of neutralizing anti-TNFα antibodies in CoMTB stimulation. The secretion of MMP-9 from neutrophils results in the destruction of type IV collagen, a major constituent of the basement membrane in the BBB, and this was inhibited by neutralizing MMP-9. This observation may explain why increased MMP-9 expression in CNS diseases corresponds to BBB disruption and is associated with neurological deficit and death [13, 29]. There are several potential targets in the MMP-9 regulatory pathway for host-directed therapy in CNS-TB, and of note, both specific and broad-based MMP-inhibitors are currently being explored as adjunctive therapy in other CNS-diseases such as autoimmune encephalomyelitis and stroke [30, 31].

We explored potential intracellular switch points in the regulation of neutrophil MMP-9 secretion and showed that the MAP-kinase, Akt-PI_3_ kinase, and NF-kB have the key roles. This is consistent with our findings of MMP regulation in other CNS cells such as astrocytes and microglial cells [32–34]. However, we observed divergent effects with *M.tb* and CoMTB stimulation on neutrophil intracellular signaling pathways. In the presence of virulent *M.tb*, neutrophil MMP-9 secretion was not suppressed by targeted chemical inhibitors of intracellular signaling paths upstream of transcription. In contrast, CoMTB, which contains cytokines including TNFα, results in neutophil MMP-9 secretion and this secretion was decreased by blockade of the p38 and ERK MAP kinases and PI3 kinase paths. This was in contrast to previous findings that TNFα-induced neutrophil MMP-9 secretion is independent of the p38 and ERK kinases [16]. We postulate the differences caused by CoMTB and *M.tb* may in part be due to *M.tb* triggering cellular necrosis in neutrophils that we and others demonstrated [35], leading to unregulated release of their proteases, and consequently, pathway modulation did not affect total MMP-9 release. This highlights the importance of monocytes in mediating neutrophil secretion of MMP-9 that drives matrix destruction in TB.

NF-kB inhibition decreased neutrophil MMP-9 secretion in response to both direct infection and monocyte-dependent networks. We have previously demonstrated that NF-kB inhibition decreased neutrophil MMP-8 secretion which drove type I collagen degradation [6], a fibril which is important in lung TB but minimally present in the central nervous system. We now demonstrate that neutrophil MMP-9, which drives destruction of type IV collagen present in the BBB, is suppressed by NF-kB inhibition. Thalidomide inhibits NF-kB by suppressing IkB kinase activity [36] and has been used to treat paradoxical reaction in CNS-TB [37, 38], but its considerable side effects such as teratogenicity and peripheral neuropathy mean that its potential as use for a host-directed therapy will be very limited.

Finally, we investigated the effects of anti-TNFα and dexamethasone on neutrophil MMP-9 secretion and found that anti-TNFα treatment significantly inhibited neutrophil MMP-9 secretion, while dexamethasone partially inhibited MMP-8 but did not affect MMP-9 secretion. The lack of response of neutrophil MMPs to dexamethasone was unexpected, given our previous finding that dexamethasone decreased CSF MMP-9 in a cohort of Vietnamese patients with CNS-TB [8]. The cells other than neutrophils such as the astrocytes, microglial, and neuronal cells also secrete MMP-9 and may contribute to the total suppression of CSF MMP-9 causing the effect that we previously observed. Glucocorticoids are established adjuncts to treatment of CNS-TB and inhibit NF-kB activity through induction of IkB synthesis, among other mechanisms, to decrease inflammatory responses and decrease MMP-9 [39]. However, steroids have significant off-target effects such as immunosuppression, steroid-induced diabetes mellitus, and Cushing’s syndrome. Anti-TNFα inhibitors are sometimes used to treat refractory paradoxical reactions in CNS-TB [25, 26] and may lead to improved neurological outcome by decreasing MMP-9 secretion.

Our study has limitations, including the use of chemical inhibitors, which may have off-target effects, to evaluate intracellular signaling pathways in neutrophils. Ideally, selective pathway inhibition using siRNA would be more specific. However, this is technically challenging in primary neutrophils as they die rapidly in vitro and hence, we and others have been unable to proceed with this approach. Also, our findings of TNFα in mediating neutrophil MMP secretion have to be carefully interpreted in clinical care as there is a potential optimal concentration for host control of infection [18]. While excessive TNFα drives paradoxical TB reactions, blocking TNFα can lead to reactivation of latent TB [15, 40], so any therapeutic intervention requires very careful evaluation.

## Conclusions

In summary, we show MMP-9 is expressed by neutrophils during the host response in CNS-TB. Neutrophil MMP-9 gene expression and secretion are driven by both direct effects of *M.tb* infection and monocyte-dependent networks. Destruction of type IV collagen, a major component of the BBB, is driven by neutrophil MMP-9. The MAP-kinase and Akt-PI_3_ kinase pathways regulate MMP-9 secretion through monocyte-dependent networks but not after direct *M.tb* infection. Neutralizing TNFα significantly suppressed neutrophil MMP-9 secretion and gene expression. Host-directed therapies that target MMPs and the key regulatory pathways should be evaluated to improve neurological and mortality outcomes in CNS-TB.

## Abbreviations

BBB: Blood-brain barrier
CNS-TB: Central nervous system tuberculosis
CoMTB: Conditioned media of monocytes infected with *M. tuberculosis*
CSF: Cerebrospinal fluid
HIV: Human immunodeficiency virus
LTA_4_: Leukotriene A_4_
*M.tb*: *Mycobacterium tuberculosis*
MAP-kinase: Mitogen-activated protein kinase
MMP: Matrix metalloproteinases
NF-kB: Nuclear factor-kB
NGAL: Neutrophil gelatinase-associated lipocalin
PCR: Polymerase chain reaction
TB: Tuberculosis
TIMP: Tissue inhibitor of metalloproteinases
TNF: Tumor necrosis factor
